# Comparison of Cytotoxicity and Genotoxicity in Three Types of Indirect Restorative Materials on Human Periodontal Stem Cells

**DOI:** 10.3290/j.ohpd.b4211055

**Published:** 2023-07-13

**Authors:** So Yeong Park, Kyung Hee Lee

**Affiliations:** a Assistant Professor, Department of Dental Hygiene, Division of Health Science, Dongseo University, Busan, Republic of Korea. Performed experiments, corrected and analysed the data, approved the submitted manuscript.; b Professor, Department of Dental Hygiene, Division of Health Science, Dongseo University, Busan, Republic of Korea. Oversaw the entire project and drafted the manuscript, approved the submitted manuscript.

**Keywords:** cytotoxicity, ERK, human periodontal stem cells, resin monomer, ROS

## Abstract

**Purpose:**

This study aimed to compare the cell toxicity and biological characteristics of Ketac GIC (glass-ionomer cement), Nexus RMGIC (resin-modified glass-ionomer cement), and RelyX RC (resin cement) in human periodontal stem cells (PDSCs).

**Materials and Methods:**

To compare the effects of Ketac GIC, Nexus RMGIC, and RelyX RC on PDSCs, the cements were diluted from 1:2 to 1:8. PDSCs were then treated with the serially diluted cements with or without N-acetyl-cysteine (NAC), and cell survival was measured using water-soluble tetrazolium salt (WST-1) assay. Intracellular reactive oxygen species (ROS) was measured using 2′,7′-dichlorofluorescin diacetate (DCFDA), and western blot analysis was performed to observe phosphorylation and activation of extracellular signal-regulated kinase (ERK) by Nexus RMGIC or RelyX RC.

**Results:**

Cell death and proliferation were dose-dependently reduced following Nexus RMGIC or RelyX RC treatment. In addition, Nexus RMGIC or RelyX RC showed an increase intracellular ROS generation compared to Ketac GIC. Pretreatment with NAC confirmed the suppression of cell toxicity and ROS generation induced by Nexus RMGIC or RelyX RC. Nexus RMGIC or RelyX RC activates ERK phosphorylation, not p38 phosphorylation, in PDSCs.

**Conclusion:**

This study showed that the treatment with Nexus RMGIC or RelyX generates intracellular ROS and cell death through the ERK signaling pathway in PDSCs. In contrast, these effects were not observed with Ketac GIC, indicating that resin-based materials may have cytotoxic and genotoxic effects on PDSCs.

One of the most severe oral conditions is periodontal disease, which has a prevalence rate of over 40% in adults.^22^ In particular, periodontal disease has steadily increased in young and middle-aged adults, leading to early tooth loss. This loss can lead to problems, such as hindrances in eating and speaking, poorer nutrition, and depression.^26,42^ Periodontal disease can also be exacerbated by oral environmental changes, such as generating intraoral reactive oxygen species (ROS).^8^ Recent studies have revealed that excessive ROS production due to external stimuli can lead to cell death, but low levels of ROS play a role in cell growth and immune function.^31^ ROS generation by pathological causes can destroy periodontal tissue and worsen chronic periodontitis. Clinical studies demonstrate that prescribing antioxidant agents to patients with periodontal disease can improve the gingival index, reduce periodontal pocket depth, and inhibit clinical attachment loss.^1^ Thus, suppressing excessive ROS production in the oral environment prevents damage to periodontal tissue. Despite the importance of controlling ROS production in the oral environment to prevent periodontal tissue damage, additional reports on effective approaches for ROS regulation are needed.

Various dental materials have been recently developed, including indirect restorative cements commonly used in prosthetic dentistry for inlays, onlays, and crowns. Newly developed resin cements are widely used in clinical practice due to their high strength and resistance to solubility in oral environments.^15^ Such resins comprise a matrix, filler, coupling agent, and initiators. The main components of the cement matrix are monomers such as triethylene glycol dimethacrylate (TEG-DMA), 2-hydroxy-ethyl methacrylate (HEMA), urethane dimethacrylate (UDMA), bisphenol-A glycidyl dimethacrylate (bis-GMA) and bisphenol A. Among them, the most abundant components of the matrix, HEMA and TEG-DMA, are released in high quantities into the intraoral environment.^43^ The continuous exposure of pulp cells to resin components through dentinal tubules induces cell death and increases ROS production.^29^ Elevated ROS levels lead to cell growth inhibition and death.^38^ HEMA or TEG-DMA induces oxidative stress and genotoxicity, in addition to altering the cell cycle.^5,16,24,34^ Additionally, HEMA has been identified as a common allergen and can thus cause allergic reactions.^14,32^ Considering the potential adverse effects, it is crucial to consider the ability of HEMA to incorporate into cell membrane lipid bilayers, be solubilised by monomers, and quickly diffuse through thin or acid-treated dentin, as these abilities could potentially increase the risk of adverse pulp reactions.^9,20^

Incomplete polymerisation of resin monomers produces redox imbalance, oxidative damage, and mitochondrial dysfunction.^2,3^ Redox system imbalance can cause excessive ROS production and reduce glutathione (GSH) levels. GSH helps prevent damage to cellular structures caused by ROS, and its depletion leads to cell damage.^33^

N-acetylcysteine (NAC) is the precursor of GSH and is a thiol-containing antioxidant that regulates intracellular redox levels. It can directly scavenge free radicals and protect cells against oxidative stress-induced death. NAC replenishes GSH levels, inhibiting the damaging effects of free radicals.^23^ Treatment with NAC can suppress cytotoxicity and genotoxicity caused by HEMA or TEG-DMA.^17,37^ Together, HEMA and TEG-DMA–mediated abnormal cell cycles are inhibited by NAC,^36^ and NAC protects against HEMA-initiated damage of dental pulp stromal cells.^27^ Furthermore, ROS is regulated by the mitogen-activated protein kinase (MAPK) signaling cascade. MAPKs are serine/threonine kinases; p38 and the extracellular signal-regulated kinases ERK1 and ERK2 activate their kinase domains.^11^ Previous studies reported that HEMA or TEG-DMA cause activation of ERK1/2 or p38 and induce cell apoptosis in pulp cells.^34^ However, the role of ERK1/2 and p38 signaling in HEMA or TEG-DMA-induced cell death differs in diverse cell types.^21,39^ Although resin monomer-mediated cell cytotoxicity has been reported, the biocompatibility of new resin composites is unclear. It is essential to study various features of dental restorative and prosthetic materials as they contact tooth structures, periodontium, and oral soft tissues. Thus, this study compares the cell toxicity and biological characteristics of Ketac GIC(glass-ionomer cement), Nexus RMGIC (resin-modified glass-ionomer cement), and RelyX RC (resin cement) in human periodontal stem cells (PDSCs).

## Materials and Methods

### Cell Culture

Human PDSCs were obtained from the periodontal ligament of first premolars extracted for orthodontic reasons and sorted by fluorescence-activated cell sorting. PDSCs were approved by the IRB (1041493-E-2020-001) of Dongseo University in Korea. The cells were cultured in α-Minimum Essential Medium (α-MEM, Gibco; Rockville, MD, USA), 10% fetal bovine serum (Gibco), and 100 μg/ml penicillin/streptomycin (Gibco). Cells were grown in 6-well culture plates at 2 x 10^5^ cells/well and cultured at 37ºC in a 5% CO_2_ incubator. In this study, we used cells from the 5th to 8th passages.

### Cell Treatment

PDSCs were plated at 2 x 10^5^ cells/well in a 6-well culture plate. After 24 h, the cells were cultured until cell density reached 60%–80%. Three dental cement types were used: Ketac GIC (glass-ionomer cement; 3M Oral Care; St Paul, MN, USA), Nexus RMGIC (resin-modified glass-ionomer cement; 3M Oral Care), and RelyX RC (resin cement; 3M Oral Care). Their compositions are shown in Table 1. The cements were mixed following the manufacturer’s protocols and placed in Ø5 x 2-mm Teflon rings. After polymerisation, the cement-filled rings were incubated in 1 ml of serum-free α-MEM medium, which is maintenance media without serum, for 24 h at 37ºC. The eluates of Ketac GIC, Nexus RMGIC, or RelyX RC were diluted to the final concentration indicated in each experiment and filtered using 0.2-µm syringe filters, ensuring sterility, and immediately added to the cells. PDSCs were seeded in 96-well plates for cytotoxicity experiments and incubated for 24 h at 37ºC under 5% CO_2_. After 24 h, the maintenance media was removed and replaced with 200 µl per well of undiluted eluate or serial dilutions of each cement eluate (up to 1:8). NAC (Sigma Aldrich; St Louis, MO, USA) was dissolved in sterilised distilled water and diluted in culture medium to 10 mM. PDSCs were pretreated with 10 mM NAC for 30 min before each cement treatment.

**Table 1:** Tested indirect restorative cements and their composition

Materials	Type	Composition*
Ketac Cement Easymix	GIC	Powder: glass powder, polycarboxylic acid, pigments Liquid: water, tartaric acid, conservation agents
Nexus Kerr RMGIC	RMGIC	HEMA(10-30%), YbF_3_, bis-GMA(1-5%), bis-EMA(1-5%)
RelyX Luting Cement	RC	Powder: FAS glass, potassium persulfate, ascorbic acid, opacifying agent Liquid: methacrylated polycarboxylic acid, water, HEMA (25-35%), tartaric acid

* According to the manufacturer, 3M Oral Care.

### Cell Viability Assay

Cells were seeded at a density of 1 x 10^4^ cells/well on a 96-well plate. After 24 h, the culture medium was removed, and eluates of the cements were added to the cells for 24 h. Cells were then washed and incubated with fresh medium, followed by the addition of 10 µl WST-1 (EZ-cytox cell viability assay kit, Daeil Lab; Seoul, Korea) for 1 h. Absorbance was measured at 450 nm in an ELISA reader (Multiskan FC, Thermo Fisher Scientific; Waltham, MA, USA). Untreated cells were used as controls, representing 100% cell viability.

### Measurement of Intracellular Reactive Oxygen Species(ROS)

PDSCs were cultured at 2 x 10^5^ cells/well in a 6-well culture plate. After 24 h, cells were incubated with each cement eluate. The cells were labeled with 2′,7′-dichlorofluorescin diacetate (DCFDA)/H2DCFDA-Cellular ROS Assay Kit (ab113851; Abcam; Cambridge, UK) following the manufacturer’s instructions to identify ROS. The fluorescence intensity was detected using an Olympus IX71 (Tokyo, Japan). Tert-butyl hydroperoxide was used as a positive control.

### Western Blotting

Cells were lysed with RIPA lysis buffer (T&I; Chuncheon; Kangwon, Korea) containing a phosphatase and protease inhibitor cocktail (Roche; Basel, Switzerland). The lysates were centrifuged at 13,000 rpm for 15 min at 4ºC to separate the supernatants. Proteins were separated by SDS-PAGE and transferred to PVDF membranes (IPVH00010; Millipore; Burlington, MA, USA). The membranes were blocked with 5% skim milk (BD Biosciences; Franklin Lakes, NJ, USA) for 1 h, and the primary antibodies were added and incubated overnight at 4ºC. The blots were analysed using anti-phospho-ERK1/2 (1:1000, Cell Signaling; Danvers, MA, USA), anti-ERK1/2 (1:1000, Cell Signaling), anti-phospho-p38 (1:1000, Cell Signaling), anti-p38 (1:1000, Cell Signaling), and anti-GAPDH (1:5000, Santa Cruz; Dallas, TX, USA) antibodies. The blots were exposed to chemiluminescence reagents (GE Healthcare Life Science; Chicago, IL, USA) to detect the specific protein bands and analysed using ImageJ software (National Institutes of Health; Bethesda, MD, USA).

### Data analysis

Data are presented as the means ± SEM from three independent experiments. All statistical data were analysed with one-way ANOVA followed by Dunnett’s for multiple comparisons, using GraphPad Prism statistical software (GraphPad Sofware; San Diego, CA, USA). Statistical significance was set at p < 0.05.

## Results

### Cell Viability of PDSCs Exposed to GIC, RelyX RC, or Nexus RMGIC

**Fig 1:**
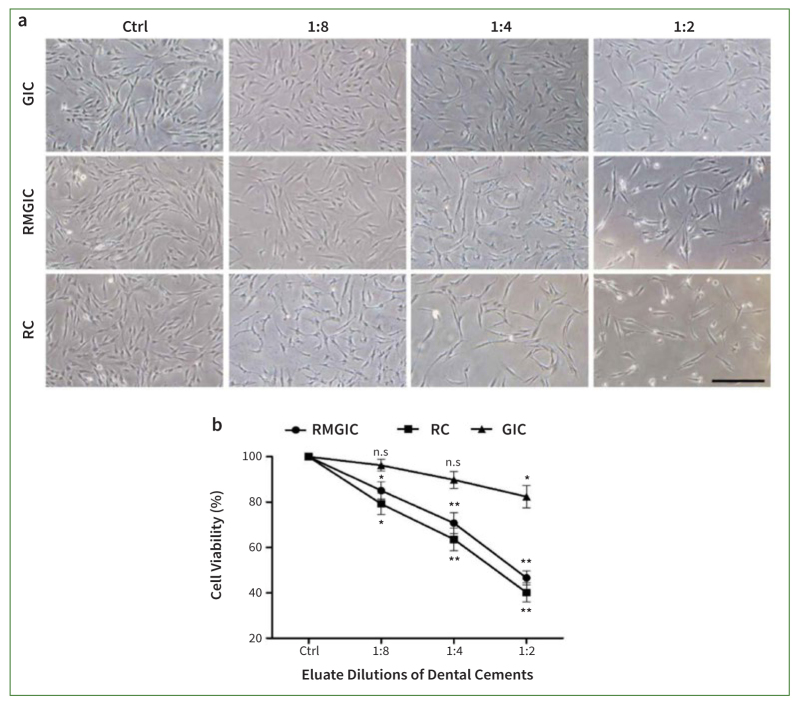
The cell viability of Ketac GIC, RelyX RC, or Nexus RMGIC on the growth of PDSCs. The images represent control, Ketac GIC, Nexus RMGIC, or RelyX RC groups after 24 h. The quantification of cell survival using the WST-1 assay as a ratio compared to the control group. The data are indicated as a percentage relative to the control group (100%), presented as the mean ± SEM from three independent experiments. One-way ANOVAs with Dunnett’s post-hoc tests. *p < 0.05, **p < 0.01. n.s.: not statistically significant compared to the control group. Scale bar: 100 μm.

The water-soluble tetrazolium salt (WST-1) assay analysed the cell survival rate of human periodontal stem cells (PDSCs) after incubation with eluates of the three types of dental cement: Ketac GIC (glass-ionomer cement), Nexus RMGIC (resin-modified glass-ionomer cement), or RelyX RC (resin cement) (Fig 1). PDSCs were treated at different concentrations (1:2, 1:4, or 1:8) of each cement for 24 h. Compared to Ketac GIC, both resin-based cements (Nexus RMGIC and RelyX RC) statistically significantly reduced cell survival rate in a dose-dependent manner. The cell survival rates of the Ketac GIC group were 96.24% ± 2.6 at 1:8, 89.81% ± 3.7 at 1:4, and 82.41% ± 5.0 at 1:2 concentrations, indicating a >80% cell survival rate even at the highest concentration of 1:2. However, a statistically significant, dose-dependent decrease in cell viability was observed in the Nexus RMGIC- or RelyX RC-treated groups (p < 0.05). In the Nexus RMGIC group, the cell survival rates were 85.12% ± 3.8 (1:8), 70.79% ± 4.6 (1:4), and 46.62% ± 3.1 (1:2), indicating that cell death increased with increasing concentration. Similar results were observed in the RelyX RC group, with cell survival rates of 79.25% ± 4.7 (1:8), 63.65% ± 4.9 (1:4), and 40.18% ± 4.3 (1:2), demonstrating statistically significant differences from the control group (p < 0.05). In addition, there were no statistically significant differences in cell survival rates between the Nexus RMGIC and RelyX RC groups (p > 0.05). Morphologically, control cells were spindle-shaped and proliferated with a specific directionality, whereas the Nexus RMGIC or RelyX RC-treated cells exhibited a fiber shrink-shaped. Thus, these materials ranked in order of causing cell toxicity are RelyX RC > Nexus RMGIC > Ketac GIC.

### Resin-based Cements Generate Intracellular ROS in PDSCs

**Fig 2:**
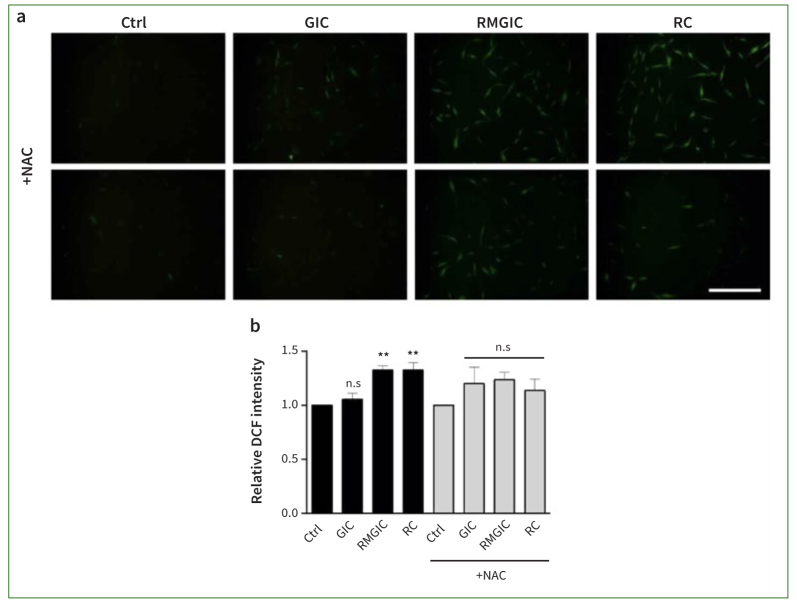
Nexus RMGIC or RelyX RC produces ROS in PDSCs. Representative images of DCFDA fluorescence (green), a ROS probe. The cells exposed to 1:8 eluates of Ketac GIC, Nexus RMGIC, or RelyX RC alone (upper images in [a]) and Ketac GIC, Nexus RMGIC, or RelyX RC following 10 mM NAC (lower images in [a]) exhibited distinct fluorescence intensities. The number of fluorescent-positive cells was counted using ImageJ. The data are presented as the mean ± SEM from three independent experiments. One-way ANOVAs with Dunnett’s post-hoc tests. *p < 0.05 and **p < 0.01. n.s.: not significant compared to the control group. Scale bar: 100 μm.

**Fig 3:**
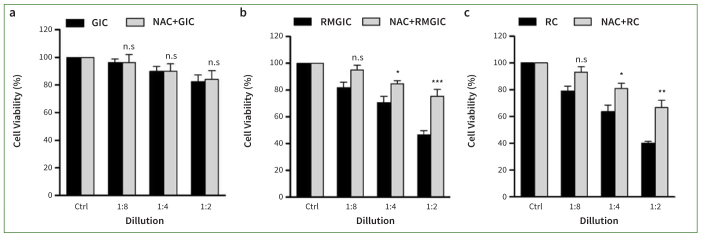
Cell viability of PDSCs after exposure to Ketac GIC, RelyX RC, or Nexus RMGIC with NAC. The WST-1 assay measured cell viability after the dose-dependent addition of Ketac GIC, RelyX RC, or Nexus RMGIC in the presence or absence of 10 mM NAC. The data are presented as mean ± SEM from three independent experiments. One-way ANOVAs with Dunnett’s post-hoc tests.*p < 0.05, **p < 0.01, ***p < 0.001. n.s.: not significant compared with control group.

To confirm whether the resin monomer generates reactive oxygen species (ROS), cells treated with Nexus RMGIC or RelyX RC were stained with DCFDA, a membrane-permeable fluorescent probe, and observed under a fluorescence microscope (Fig 2). The production of ROS was observed after treating PDSCs with Ketac GIC, Nexus RMGIC or RelyX RC. Then, the level of cellular ROS induced by the cements was measured with and without the ROS scavenger NAC. The concentration of 1:8 completely prevented cell death by NAC (Fig 3). As shown in Fig 2a, there was no difference in DCFDA fluorescence expression between the Ketac GIC-treated group and the control (p > 0.05). The intensity of DCFDA was 1.05 for the Ketac GIC group, 1.32 for the Nexus RMGIC group, and 1.33 for the RelyX RC group, relative to the control group. The Nexus RMGIC and RelyX RC groups showed statistically significant differences in intensity compared to the control group (p < 0.05); however, the difference between the Nexus RMGIC and RelyX RC groups was not statistically significant (p > 0.05). The Nexus RMGIC- and RelyX RC-treated groups showed increased DCFDA fluorescence expression, which was not observed in the group pretreated with NAC. The intensity levels with NAC pretreatment in the three groups were 1.19 for the Ketac GIC group, 1.24 for the Nexus RMGIC group, and 1.14 for the RelyX RC group. No statistically significant differences were found compared to the control group (p > 0.05), confirming that resin monomers caused ROS generation in PDSC cells. These results suggest that ROS plays a vital role in cell death induced by Nexus RMGIC or RelyX RC in PDSCs.

### Suppression of Cell Death of Resin-based Materials by NAC

To investigate whether the generation of ROS is involved in resin monomer-mediated cell cytotoxicity, we pretreated with N-acetylcysteine (NAC), a ROS scavenger. We then administered the three cement types to assess the cell survival rate using the WST-1 assay (Figs 3a–3c). Pretreatment with 10mM NAC on PDSCs statistically significantly inhibited the cell cytotoxicity caused by Nexus RMGIC or RelyX RC in a dose-dependent manner (p < 0.05). In particular, the Nexus RMGIC at 1:4 or RelyX RC-treated group at 1:4 or 1:2 showed 71.79% ± 5.3, 63.65% ± 4.9, or 40.18% ± 1.4 of cell viability rate. The presence of NAC with the Nexus RMGIC at 1:4 or RelyX RC-treated groups at 1:4 or 1:2 exhibited 84.76% ± 2.4, 80.96% ± 4.0, or 68.43% ± 6.1 of cell viability rate. Cell viability for Nexus RMGIC and RelyX RC was restored with NAC by approximately 13%-28% among PDSCs. Cell viability for the Nexus RMGIC and RelyX RC groups at a concentration of 1:8 were 81.78% ± 4.05 and 79.85% ± 5.32, respectively. Meanwhile, NAC pretreatment resulted in cell viability of 94.98% ± 3.52 and 93.10% ± 4.05, respectively, indicating that NAC inhibited cell toxicity induced by resin-based materials. However, compared to the control group, this was not statistically significant (Figs 3b and 3c, p > 0.05). In total, Nexus RMGIC and RelyX RC stimulated ROS production in PDSCs. Additionally, ROS scavenging by NAC inhibited the degree of cell death caused by the resin.

### Resin Monomer-dependent ROS Generation Induces Activation of ERK1/2

**Fig 4:**
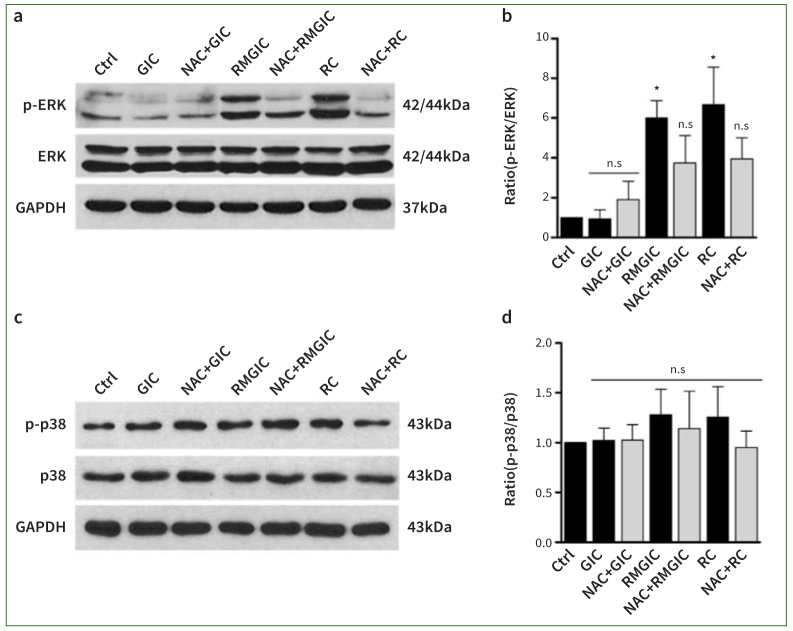
Activation of ERK1/2 results in ROS generation in PDSCs. PDSCs were incubated with 1:8 eluates of Ketac GIC, Nexus RMGIC, or RelyX RC in the presence or absence of 10 mM NAC. The cell lysates were immunoblotted with antibodies against p-ERK1/2, ERK1/2, p-p38, p38, and GAPDH. Protein levels of p-ERK1/2 or p-p38 were normalized to ERK1/2 or p38. GAPDH served as a loading control. The data are presented as mean ± SEM from three independent experiments. Comparisons between two groups of the blots were conducted by Student’s t-test. *p < 0.05, ** p < 0.01 compared with control group.

We next determined whether resin cement-generated ROS activated ERK1/2 or p38. The phosphorylation of ERK1/2 and p38 was observed in PDSCs treated with Ketac GIC, Nexus RMGIC, or RelyX RC. Additionally, the Nexus RMGIC or RelyX RC cement activated the phosphorylation of ERK1/2 compared to the Ketac GIC group (Figs 4a and 4b) (p < 0.05). This phosphorylation was statistically significantly inhibited in the group pretreated with NAC, confirming that the ROS increase by Nexus RMGIC or RelyX RC occurs through the ERK1/2 signaling pathway. However, phosphorylation of p38 was not activated by Nexus RMGIC or RelyX RC treatment. Thus, Nexus RMGIC- or RelyX RC-mediated ROS generation activated ERK – but not p38 – signaling (Figs 4c and 4d) (p > 0.05).

## Discussion

Various dental materials are available with improved aesthetics and user friendliness, but have limited biocompatibility in the oral environment and potentially harmful mechanisms. Residual monomers of resin composites can easily circulate in saliva. They can be absorbed by the skin, oral mucosa, enamel, dentin, bone, and connective tissue for extended periods, leading to cytotoxic reactions.^18^ Additionally, the exposed resin matrix can cause contact dermatitis, oral burning, and allergic reactions, resulting in adverse clinical outcomes when used as a permanent adhesive.^14,32^ Therefore, this study analysed the biological characteristics of three cement types used as indirect restorative adhesives – Ketac GIC (glass-ionomer cement), RelyX RC (resin cement), and Nexus RMGIC (resin-modified glass-ionomer cement) – to confirm the stability of Ketac GIC and determine any adverse effects of RelyX RC or Nexus RMGIC in human periodontal stem cells (PDSCs).

We observed biochemical evidence that resin monomers cause cell cytotoxicity via generating ROS and initiating a signaling cascade in PDSCs. Resin monomers have been reported to induce cell toxicity and generate ROS.^5,16,24,34^ This study examined the molecular characteristics of currently used resin-based materials and compared their cytotoxicity risk with that of glass-ionomer cement. We compared the cytotoxicity and genotoxicity of three cements manufactured by 3M Oral Care: Ketac GIC, Nexus RMGIC, and RelyX RC. The manufacturer of Nexus RMGIC and RelyX RC have withheld the exact percentages of their HEMA compositions, although they contain up to 30% HEMA, unlike Ketac GIC, which does not contain HEMA (Table 1). Exposure of dental pulp cells to resin monomers results in decreased cell numbers.^17^ Their contact with HEMA components also damages the cell cycle and inhibits growth.^13^ Additionally, the cytotoxicity of Ketac GIC, Nexus RMGIC, and RelyX RC was tested at different concentrations on periodontal ligament stem cells. The degree of cell death was highest in the RelyX RC group, followed by the Nexus RMGI group, and was statistically significantly different from that in the control group at all concentrations. However, except for the 1:2 concentration, the Ketac GIC group did not show a statistically significant difference from the control group. This observation is consistent with reports indicating that resin cement with high contents of resin monomers induces the highest levels of cell toxicity in NIH/3T3 and L929 fibroblast cell line and that the HEMA rate is one of the critical factors in cell death.^5,35^ HEMA causes severe cytogenic effects by inducing mitotic arrest^4^ and has been shown to diffuse rapidly across dentin due to its low molecular weight and high solubility in water.^6^ Thus, the HEMA content may explain the difference in cell viability between Nexus RMGIC and RelyX RC.^7^

The low cell survival rate caused by resin monomers is also related to the intracellular ROS level. Cell cytotoxicity caused by other resin monomers, including 4-META, MDP, bis-GMA, acid monomer, and TEG-DMA has also been reported.^40^ The bis-GMA, TEG-DMA, and HEMA exposure resulted in cell toxicity in mouse fibroblasts. These monomers, including HEMA, TEG-DMA and bis-GMA, induce excessive ROS production and GSH depletion, inhibiting cell differentiation and inducing death in human dental pulp cells.^10,41^ However, treatment with N-acetyl-cysteine (NAC) inhibits cell death and ROS overproduction caused by resin monomers.^17^ Additionally, as HEMA-induced apoptosis is inhibited by GSH synthesis, GSH is involved in HEMA-induced cell death.^19^ NAC is a precursor of GSH synthesis and acts as an antioxidant by directly removing ROS. Therefore, the antioxidants such as vitamin A, C, E, and NAC are expected to reduce cell toxicity by inhibiting ROS generation.^44^ NAC, unlike vitamins E and C, effectively inhibited HEMA-induced cell death in dental pulp stromal cells, demonstrating distinct mechanisms of action.^28^ Our results demonstrated that exposure to Nexus RMGIC or RelyX RC led to approximately 30% higher intracellular ROS levels than did the Ketac GIC group, as measured by 2′,7′-dichlorofluorescein diacetate (Fig 2). However, the presence of NAC suppressed the cell death rate (Fig 3) and ROS production induced by Nexus RMGIC or RelyX RC (Fig 2). These findings are consistent with the hypothesis that the HEMA component, which is present in both Nexus RMGIC and RelyX RC, is the primary source of these effects, rather than other resin monomers, such as bis-GMA and TEG-DMA.

Cellular toxicity induced by resin monomers is associated with ROS signaling. Activation of ERK1/2 signaling by HEMA or TEG-DMA exposure remains controversial. Previous studies have demonstrated that HEMA exposure induces ERK1/2 and p38-mediated cell death in salivary-gland cell lines.^34^ In contrast, TEG-DMA induces cell death by activating not ERK but PI3K signaling in pulp cells.^39^ These signaling disparities may be attributed to variations in cell lines andmaterial composition. Here, Nexus RMGIC and RelyX RC contain a high quantity of HEMA and are expected to activate the ERK1/2 signaling cascade (Fig 4), consistent with the immunoblotting results by Samuelsen et al.^34^ However, the dissimilarity from other studies is due to the variation in sensitivity of ERK1/2 to oxidative signals among cell types.^25^

Here, three indirect restorative adhesives – Ketac GIC, Nexus RMGIC, and RelyX RC – were used to compare the cytotoxic and genotoxic properties of resin-based materials. The results showed that treatment with Nexus RMGIC or RelyX RC generates intracellular ROS and cell death through the ERK signaling pathway in PDSCs. In contrast, these effects were not observed with Ketac GIC, indicating that resin-based materials may have cytotoxic and genotoxic effects on PDSCs. Cell toxicity induced by four self-adhesive dual-curing resin cements, including RelyX RC, was significant in human mesenchymal stem cells (hMSC) after 24 h,^12^ consistent with the results of this study. In addition, differences in the release rates of resin monomers, such as TEG-DMA and bis-GMA, have been reported at 24 h and longer durations (7 and 21 days).^30^ Accordingly, further studies will be needed to confirm the effects of HEMA over a longer term. Furthermore, it is necessary to apply RelyX RC or Nexus RMGIC only to localised areas, and the safety of various resin composites used in clinical dentistry must be evaluated. As resin comes into direct contact with periodontal tissues, further research is necessary to determine the cellular mechanisms and the degree of cytotoxicity.

## Conclusion

Exposure to resin-based materials, including a resin-modified glass-ionomer cement and a resin cement, showed increased ROS-mediated cell cytotoxicity and phosphorylation of ERK, while these reactions were not observed with the glass-ionomer cement. These findings suggest the potential of resin-based materials to cause cytotoxicity and act as allergens in cells when used in clinical dentistry, emphasising the importance of investigating their biocompatibility.
